# Molecular Crosstalk and Therapeutic Synergy: Tyrosine Kinase Inhibitors and Cannabidiol in Oral Cancer Treatment

**DOI:** 10.3390/cimb47080584

**Published:** 2025-07-23

**Authors:** Zainab Saad Ghafil AlRaheem, Thao T. Le, Ali Seyfoddin, Yan Li

**Affiliations:** 1 School of Science, Faculty of Health and Environment Science, Auckland University of Technology, Auckland 1010, New Zealandali.seyfoddin@aut.ac.nz (A.S.); 2 Maurice Wilkins Centre, Auckland 1010, New Zealand

**Keywords:** head and neck squamous cell carcinoma (HNSCC), epidermal growth factor receptor (EGFR), tyrosine kinase inhibitors (TKIs), cannabidiol (CBD)

## Abstract

Head and neck squamous cell carcinoma (HNSCC) is the sixth most common malignancy worldwide, with oral squamous cell carcinoma (OSCC) accounting for a significant portion of cases. Despite advancements in treatment, only modest gains have been made in HNSCC/OSCC control. Epidermal growth factor receptor (EGFR) tyrosine kinase inhibitors (TKIs) have emerged as targeted therapies for OSCC in clinical trials. However, their clinical efficacy remains a challenge. Cannabidiol (CBD), a non-psychoactive phytochemical from cannabis, has demonstrated anticancer and immunomodulatory properties. CBD induces apoptosis and autophagy and modulates signaling pathways often dysregulated in HNSCC. This review summarizes the molecular mechanisms of EGFR-TKIs and CBD and their clinical insights and further discusses potential implications of combination targeted therapies.

## 1. Introduction

**Figure 1:**
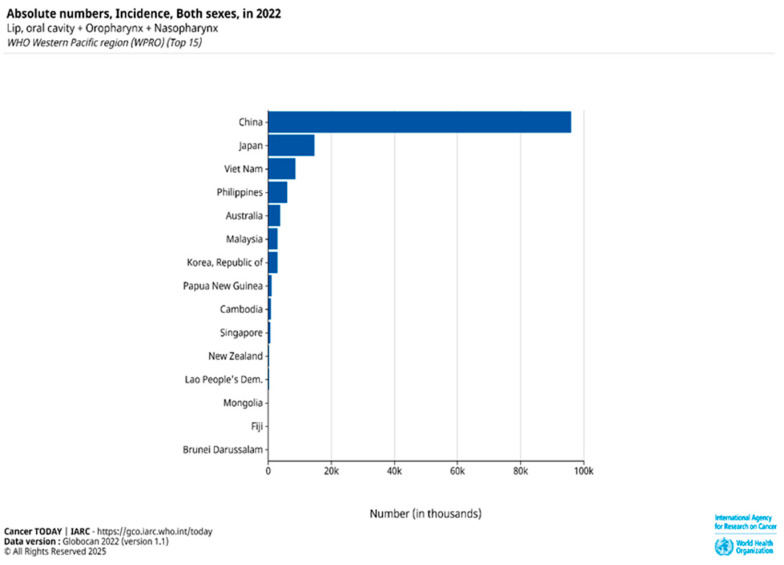
Absolute number incidence of lip, oral cavity, oropharyngeal, and nasopharyngeal cancers in both sexes across the top 15 countries in the WHO Western Pacific Region (WPRO) based on GLOBOCAN 2022 data [1].

Head and neck squamous cell carcinoma (HNSCC), ranked as the sixth most common cancer globally, is predominantly composed of OSCC [1,2,3]. The disease is responsible for approximately 450,000 deaths worldwide each year, highlighting its significant global health burden. According to GLOBOCAN 2022 estimates from the International Agency for Research on Cancer (IARC), Papua New Guinea, Southeast Asian countries, and Australia have some of the highest age-standardized incidence rates (ASIRs) of HNSCC globally, with ASIRs of 17.5, 9–10, and 8.3 per 100,000, respectively [1]. While the rising occurrence of HPV-related oropharyngeal infections has driven the increased prevalence of HNSCC in the United States and Western Europe, the elevated incidence of HNSCC in Southeast Asia and Australia/New Zealand is linked to the use of particular products containing carcinogens [3,4]. In the Asia-Pacific region, lip, oral cavity, oropharyngeal, and nasopharyngeal cancers continue to pose significant health challenges due to their high absolute incidence rates (Figure 1).

Despite advances in standard treatments and progress in research, only modest gains have been made in HNSCC/OSCC control [5]. Advanced-stage OSCC has a poor prognosis, with a 5-year survival rate of less than 30% in unresectable or metastatic cases due to the limited effectiveness of current therapies. Emerging clinical evidence suggests epidermal growth factor receptor (EGFR)–tyrosine kinase inhibitors (TKIs), either alone or in combination with other therapies, such as chemotherapy, radiotherapy, or immunotherapy (e.g., nivolumab or pembrolizumab), have shown promise as targeted regimens for head and neck cancers, including oral cancers [4,5]. However, patients with mutations in the phosphatidylinositol 3-kinase (PI3K) pathway have shown poor response to irreversible EGFR-TKIs because these mutations activate signals that continue to promote cancer cell growth, even when EGFR is blocked [6,7].

Cannabidiol (CBD) has been shown to inhibit PI3K/Akt signaling, induce apoptosis and autophagy, and exhibit anti-inflammatory and anti-angiogenesis properties [8]. CBD’s ability to modulate signaling pathways, reduce inflammation, and induce cancer cell death could complement the targeted action of TKIs [9]. This synergy could potentially enhance the effectiveness of TKIs in eradicating cancer cells and thus prevent multidrug resistance [9,10,11,12]. Coordinated targeting of EGFR via CBD and TKIs could represent a logical approach for cancer treatment, particularly in HNSCC, where CBD receptors and EGFR signaling pathways interact [13]. Although this review focuses on the potential synergy between CBD and EGFR-TKIs, it is important to note that CBD’s anticancer effects are not limited to EGFR inhibition.

Preclinical studies suggest that CBD can also influence other receptor tyrosine kinases (RTKs), such as HER2, VEGFR, and PDGFR, which are involved in tumor growth, angiogenesis, and metastasis. CBD has been reported to inhibit HER2-driven tumor cell proliferation by binding to the HER2 kinase domain, thereby reducing activation of the downstream PI3K/AKT and MAPK/ERK pathways, which are critical for cancer cell survival [14]. Furthermore, CBD also suppresses VEGF signaling by reducing hypoxia-inducible factor 1-alpha (HIF-1α) expression and preventing VEGFR activation on endothelial cells, ultimately inhibiting angiogenesis [15]. Moreover, CBD interferes with PDGFR activity and downstream signaling pathways, like PI3K/AKT and MAPK/ERK, both of which contribute to cancer cell migration and resistance mechanisms. Since PDGFR is active not only in tumor cells but also in the tumor microenvironment, its inhibition by CBD may disrupt stromal support and reduce tumor progression [16].

Combinatorial therapies are increasingly being explored in cancer treatment to overcome drug resistance, boost therapeutic efficacy, and minimize toxicity. The incorporation of CBD into oral cancer therapy holds promise for improving patient outcomes by alleviating treatment-induced side effects and symptoms. CBD exhibits multiple pharmacological properties, including anticonvulsant, neuroprotective, antioxidant, analgesic, and anti-nausea effects. CBD also significantly lowers muscular tension, restlessness, fatigue, and cognitive impairments, such as difficulty concentrating. The U.S. Food and Drug Administration (FDA) has approved two cannabinoid-based oral medications, dronabinol (Marinol) and nabilone (Cesamet), specifically to relieve nausea and vomiting caused by cancer chemotherapy, underscoring their medical usefulness in oncology [17]. Furthermore, the novel combination strategy may enhance treatment outcomes in oral cancer by mitigating resistance to chemotherapeutic agents. Drug resistance remains a major clinical challenge contributing to therapeutic failure in cancer patients. Notably, CBD has demonstrated the ability to inhibit the growth and metastasis of cisplatin-resistant non-small cell lung cancer (NSCLC) [18], highlighting its potential to overcome drug resistance in other cancer types. Collectively, these contributions have the potential to substantially enhance patient outcomes, positioning this research as a pivotal advancement in the field of oncology.

This review summarizes the molecular mechanisms of EGFR-TKIs and CBD and their clinical insights and further discusses potential implications of combination targeted therapies.

## 2. Overview of Oral Cancer

The major constituent of head and neck cancers is OSCC, which is derived from the mucosal epithelium in the oral cavity, pharynx, and larynx and is known collectively as HNSCC [3,19]. OSCC accounts for more than 90% of head and neck cancers, which include malignancies of the nasal cavity, oral cavity, paranasal sinuses, nasopharynx, hypopharynx, and larynx [20,21,22]. The most common intraoral location of the OSCC is the tongue, followed by the floor of the mouth (which is the second common site), while the gingival, buccal mucosa, labial mucosa, and hard palate are less common sites [23].

The main risk factors for oral cancer include smoking, alcohol consumption, betel quid use, and overexposure to sunlight, which have been particularly associated with cancers of the skin and lip [24]. Accumulating evidence suggests that alcohol consumption is a predominant risk factor, followed by tobacco [3,21]. Further investigations are needed to determine the prevalence of oral malignancies attributed to oncogenic human papillomavirus (HPV) infection [19]. The interaction between these factors and viral infection in the situation of oral malignancy is a subject of ongoing investigations.

Certain high-risk HPVs are etiological factors for oral cancer; about 20% of OSCC are positive for HPVs, especially HPV-16 and -18 [25]. HPV types such as HPV-16 and -18 can integrate their genomes into the host DNA, leading to the expression of E6 and E7 oncogenes, which deregulate key molecules involved in the cell cycle [26,27]. Furthermore, the oncogenic proteins E6 and E7 inactivate tumour suppressors p53 and pRb, respectively [28]. Many clinical studies have shown that HPV status is an independent prognostic factor, with better survival rates observed in HPV-positive individuals compared to those who are HPV-negative [29]. Subsequently, HNSCC with positive HPV is more responsive to radiotherapy, and OPC with HPV has a better prognosis than conventional squamous cell carcinoma (SCC). This is because HPV-positive tumors usually have fewer genetic changes and are more sensitive to radiation. They also tend to trigger a stronger immune response, which helps improve treatment outcomes [30,31]; however, their association with the prognosis of OSCC has not been well-documented [32].

The WHO predicts a global increase of approximately 20% in OSCC incidence over the coming decades [33,34]. It is important to mention that the incidence of oropharyngeal malignancies has increased in many countries, including those in Europe [35], as well as Japan [36], South Korea [37], Brazil [38], Canada [39], the United States [40], England [41], Denmark [42], Australia [43], Scotland [44], and New Zealand [45].

**Figure 2:**
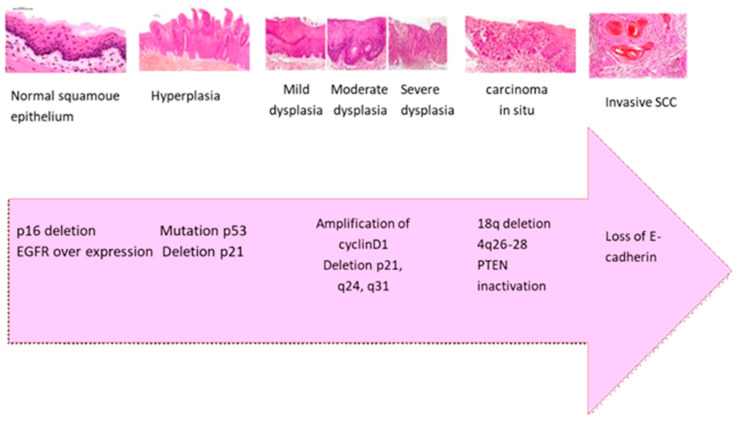
The transition from normal squamous epithelium to invasive SCC involves sequential morphological changes (including dysplasia, carcinoma in situ, and invasive carcinoma, magnification 400×) accompanied by key molecular events such as p16 deletion, EGFR overexpression, TP53 mutation, cyclin D1 amplification, and PTEN inactivation. These alterations cause malignant transformation, enhanced proliferation, and loss of cellular adhesion, particularly E-cadherin, contributing to tumor invasiveness and metastasis [23,46,47].

Current scientific evidence indicates that the transition from normal epithelium to oral carcinoma results from the accumulation of genetic and epigenetic changes over time. These changes include the inactivation of tumor suppressor genes (TSG) (e.g., *TP53*, *CDKN2A/p16*, *ING1,* and *ING3*) [46], the activation of oncogenes (e.g., *EGFR/erbB1*, *ERBB2/HER2*), and mutations in DNA mismatch repair genes (e.g., *MLH1, MSH2, MSH6*, *PMS2*) [47,48]. As illustrated in Figure 2 and summarized in Table 1, this development follows a sequence of histopathological stages starting with normal squamous epithelium and advancing through hyperplasia, mild to severe dysplasia, carcinoma in situ, and ultimately SCC. Specific molecular changes accompany each stage. Early in the process, p16 deletion and EGFR overexpression are commonly observed. As dysplasia progresses to a more severe stage, *TP53* mutations and p21 deletions contribute to cell cycle dysregulation. In carcinoma in situ, there is often amplification of cyclin D1 and chromosomal deletions at q24 and q31, promoting cellular proliferation. As the lesion progresses to invasion, PTEN inactivation and deletions on 18q and 4q26–28 further disrupt tumor suppressor functions. Finally, the loss of E-cadherin impairs cell–cell adhesion [23,46,47].

HNSCC is typically addressed through initial surgery if the tumor is resectable, followed by adjuvant therapy that may involve radiation and, if necessary, chemotherapy [49,50]. For early-stage tumors (stages I and II) in the oral cavity, the primary approaches include either surgery or definitive radiation therapy. On the other hand, locoregionally advanced cancers (stages III and IVa,b) in the oral cavity are managed with surgery, followed by adjuvant radiation therapy, with or without concurrent chemotherapy [50]. About two-thirds of HNSCCs manifest in advanced stages, encompassing both local stages (III/IVa/IVb) and the metastatic stage (IVc) [51].

The effectiveness of surgery is hindered by limitations related to gender, age, and health condition. To address this limitation, standard chemotherapy agents, like cisplatin and 5-fluorouracil (5-FU), are employed alongside radiotherapy. However, this approach is restricted by prevalent resistance, lack of specificity, and undesirable side effects, including nephrotoxicity, mucositis, and fatigue, particularly in older patients [52,53].

In recent years, increasing rates of local recurrence and distant metastasis have created a bottleneck in improving survival outcomes for patients with HNSCC. To overcome this challenge, there is a pressing need for the development and integration of molecularly targeted antibodies and immunotherapies that offer high efficacy, strong selectivity, low toxicity, and the ability to counteract resistance to conventional treatments, such as radiotherapy and chemotherapy [52,53,54].

As summarized in Table 2, EGFR-targeted therapies for HNSCC include both monoclonal antibodies and TKIs. Monoclonal antibodies such as cetuximab, panitumumab, zalutumumab, and nimotuzumab bind to the extracellular domain of EGFR, blocking ligand binding and receptor activation while also promoting antibody-dependent cellular cytotoxicity (ADCC). In contrast, EGFR-TKIs like gefitinib, erlotinib, lapatinib, afatinib, and dacomitinib inhibit the intracellular tyrosine kinase domain of EGFR, thereby preventing downstream signaling pathways, such as PI3K/AKT and MAPK/ERK, which drive tumor proliferation and survival.

Immunotherapy combinations are being actively investigated in Phase III trials to enhance efficacy and reduce toxicity in HNSCC. These combinations primarily involve immune checkpoint inhibitors, such as PD-1 inhibitors, like pembrolizumab and nivolumab, or PD-L1 inhibitors, such as avelumab and durvalumab, used either alone or in combination with other chemotherapy, radiotherapy, EGFR-targeting antibodies or other immunotherapeutic agents [54]. Notably, a recent Phase III trial (KEYNOTE-689 trial (NCT03765918)) exploring the addition of an innovative neoadjuvant and adjuvant pembrolizumab in resectable HNSCC has demonstrated significantly improved event-free survival [55].

EGFR antibody–immunotherapy approaches, which combine EGFR-targeting antibodies, like cetuximab, with immune checkpoint inhibitors, are also under investigation to enhance immune activation and overcome resistance [56]. Cetuximab, an anti-EGFR IgG1 monoclonal antibody, not only inhibits EGFR-mediated signaling but also engages immune effector functions, such as antibody-dependent cellular cytotoxicity (ADCC), thereby providing a rationale for combination with immunotherapy [56]. For instance, the GORTEC 2017-01 (REACH) Phase III trial (NCT02999087) is evaluating the efficacy and safety of combining cetuximab and avelumab (anti-PD-L1) with radiotherapy versus standard radiotherapy combinations in locally advanced squamous cell carcinoma of the head and neck (LA-SCCHN) [57].

Furthermore, novel targeted agents, such as antibody drug conjugates (ADCs), are designed to deliver cytotoxic payloads directly to tumor cells with high specificity. Emerging ADC formats involving bispecific ADCs, dual-drug ADCs, and conditionally activated probody drug conjugates are being developed to improve tumor targeting while reducing toxicity [58]. For example, MRG003, an anti-EGFR antibody–drug conjugate, is currently being evaluated in an ongoing Phase III clinical trial (NCT05751512) to assess its efficacy and safety compared to cetuximab or methotrexate in patients with recurrent/metastatic (R/M) HNSCC who have progressed on prior treatments [59]. The novel targeted agent PDS0101, a therapeutic vaccine that targets HPV16 E6/E7 oncoproteins, is currently being evaluated in the Phase III VERSATILE-003 trial (NCT04260126). Administered in combination with pembrolizumab, this vaccine has demonstrated promising results in earlier trials and represents a targeted immunotherapeutic strategy for HPV+HNSCC [60].

## 3. Epidermal Growth Factor Receptor (EGFR)

EGFR is defined as a family of receptor tyrosine kinases (RTKs) that form a subset of tyrosine kinases crucial for cell communication and governing various intricate biological processes such as cellular proliferation, movement, specialization, and metabolic activities. Human biology encompasses 58 identified RTKs, all exhibiting a uniform protein architecture consisting of an extracellular ligand-binding domain, a solitary transmembrane helix, and an intracellular segment housing a regulatory region adjacent to the membrane, a tyrosine kinase domain (TKD), and a C-terminal tail [61].

The EGFR family consists of four distinct receptors, including the epidermal growth factor receptor (also known as ErbB-1/HER1), ErbB-2 (neu, HER2), ErbB-3 (HER3), and ErbB-4 (HER4) [62]. EGFR is triggered by binding to specific ligands such as epidermal growth factor (EGF), transforming growth factor-alpha (TGF-alpha), amphiregulin (AREG), epigen, β-cellulin, and heparin-binding EGF(HB-EGF). Ligand binding to domains I and III of the RTK induces the EGFR for a conformational alteration that causes the exposure of the dimerization loop in domain II of the receptor. As a result of this exposure of the dimerization loop, the receptor at the plasma membrane undergoes homodimerization and heterodimerization [62,63]. This interaction stimulates the RTK, leading to phosphorylation of the intracellular domain, and mediates the interaction between the receptor and downstream effectors, such as Shc1 and Grb2 [64]. This further activates downstream signaling pathways, such as PI3/Akt (survival and apoptosis evasion) and Ras-Raf-MEK-MAPK (proliferation) [65]. HER3 is the only family member without intrinsic kinase activity [63].

EGFR is also present in healthy tissues but is upregulated in malignancies such as breast cancer, glioblastoma, and SCC [66]. In HNSCCs, high EGFR expressions are positively associated with earlier relapse, shorter disease-free survival, and reduced overall survival compared to patients with lower EGFR expression levels [67,68]. EGFR overexpression is associated with poor outcomes, the inhibition of apoptosis, and increased metastatic potential [53,59]. Notably, EGFR overexpression is observed in approximately 90% of the HNSCC cases, making it a negative prognostic marker [62,68].

Given these associations, current therapeutic strategies for HNSCC focus on EGFR inhibition. EGFR inhibition agents function by either blocking the ligand binding at the extracellular domain of EGFR (e.g., cetuximab, panitumumab, zalutumumab, and nimotuzumab) or hindering EGFR autophosphorylation in the cytoplasmic domain by competing with ATP (e.g., gefitinib, erlotinib, lapatinib, afatinib, and dacomitinib). Such interferences consequently disrupt the downstream cell signaling cascade [67]. In Section 4, a brief explanation of TKIs and their mechanism of action will be provided. Molecular targeted therapies, such as gefitinib and afatinib, are being used in tongue cancer; therefore, further details about gefitinib will be briefly discussed.

## 4. EGFR–Tyrosine Kinase Inhibitors

### 4.1. Gefitinib

Gefitinib (ZD1839 or Iressa) is a widely used EGFR-TKI. According to preclinical in vitro research, EGFR targeting with gefitinib leads to decreased cell survival, proliferation, and migration with sensitivity to the drug. Depending on the cancer cell type and the presence or absence of a sensitizing EGFR mutation, gefitinib exhibited half-maximal inhibitory concentrations (IC_50_) ranging from submicromolar levels to 13 µM across various non-small cell lung cancer (NSCLC) cell lines [69].

In early clinical trials, patients with a wide range of solid tumor types, including head and neck, lung, colon, breast, and prostate cancers, have revealed that gefitinib is generally well tolerated [70]. Moreover, it is well established that gefitinib inhibits solid tumors in vivo and induces autophagy [71,72]. This drug has been used to treat many types of cancer with EGFR mutations effectively [73]. In 2003, the FDA approved gefitinib as the first-line treatment for patients with EGFR mutation-positive advanced non-small cell lung cancer (NSCLC) [74].

Several different EGFR mutations have been discovered, although they are rare in HNSCC patients. These mutations tend to concentrate around the active site crevice of the tyrosine kinase domain. In exon 18, point mutations within the nucleotide binding loop have been identified, including substitutions at glycine 719 (Gly719), which may be replaced by serine, cysteine, or alanine [75]. In exon 19, minor deletions are observed, most notably the deletion of residues 746–750, which is common in lung cancer but rarely reported in HNSCC [75,76]. Exon 20 mutations include insertions that can enhance kinase activity, though they are infrequent in HNSCC [75]. In exon 21, the L858R point mutation, which substitutes leucine 858 with arginine, is the most prevalent EGFR mutation in lung cancer, accounting for around 40% of all cases, but it is rarely seen in HNSCC [75,76]. These mutations enhance receptor activation by stabilizing the kinase domain in its active conformation, which facilitates dimerization [61,77]. Many studies have demonstrated that lung cancer patients with EGFR kinase domain mutations benefit from EGFR-TKI treatments, while those with wild-type EGFR do not show similar advantages [61]. However, in a small-scale HNSCC clinical study, an EGFR mutation involving a deletion in exon 19 was associated with a poor response to cetuximab treatment and worse prognosis [78], as shown in Table 3.

Gefitinib has been investigated in HNSCC cell lines, where it has been frequently shown to delay tumor growth and enhance apoptosis [79,80]. This has been attributed to its inactivation of functional ERK and AKT signaling pathways in either unstimulated or EGF-stimulated conditions [80]. However, a meta-analysis suggested that gefitinib did not significantly improve the progression-free survival (PFS), overall survival rate (OSR), or objective response rate (ORR) in patients with advanced HNSCC [51]. Similarly, unfavorable results were observed in a randomized study comparing gefitinib to methotrexate in recurrent and/or metastatic HNSCC, where the EGFR gene copy number did not predict a survival advantage in individuals treated with the EGFR-TKI. Intriguingly, the response to TKIs in a specific subset of HNSCC might be attributed to mutations in ErbB2 rather than EGFR, although this initial observation awaits further validation [81].

### 4.2. Afatinib

Afatinib (BIBW 2992, Gilotrif™) is a second-generation, orally administered TKI that irreversibly targets members of the ErbB receptor family, including EGFR, HER2, and HER4 [82]. It is FDA-approved as a first-line treatment for metastatic NSCLC with common EGFR mutations [83].

Mechanistically, afatinib is an irreversible pan-ErbB TKI that covalently binds to the kinase domains of EGFR, HER2, and HER4, leading to sustained inhibition of their enzymatic activity. This covalent binding blocks both ligand-dependent and ligand-independent receptor activation, preventing autophosphorylation and transphosphorylation events within ErbB dimers. As a result, downstream signaling pathways such as PI3K/AKT, RAS/RAF/MEK/ERK, and JAK/STAT are suppressed, reducing cell proliferation, survival, and angiogenesis [84]. Afatinib also inhibits receptor heterodimerization and crosstalk, which are key mechanisms of resistance in tumors. Importantly, afatinib retains activity against resistant EGFR mutation T790M in NSCLC, activating truncation variants, like EGFRvIII, occasionally identified in HNSCC [82,84,85,86].

Afatinib has demonstrated lower IC_50_ values compared to gefitinib across EGFR-amplified cell lines, including those resistant to gefitinib [84,85]. It also shows activity in HER2-amplified breast cancer cell lines resistant to trastuzumab and lapatinib [82]. In HNSCC, where erlotinib and gefitinib have shown limited efficacy as monotherapies, afatinib has emerged as a promising drug due to its ability to inhibit the ErbB family and its encouraging preclinical and early clinical studies [85,87]. Recent clinical trials support the effectiveness of afatinib. For example, the UPSTREAM trial reported improved progression-free survival in biomarker-selected HNSCC patients, particularly those with EGFR amplification, HER2 mutations, or PTEN-high tumors [88]. Additionally, the ALPHA study demonstrated an improved ORR when afatinib was combined with pembrolizumab in platinum-refractory HNSCC [89].

## 5. Clinical Trials of Targeted Therapies in HNSCC

EGFR is overexpressed in 90% of HNSCC patients, despite the absence of active EGFR mutations [85,90]. Several clinical trials have investigated the safety and efficacy of TKIs and immunotherapies in HNSCC. Table 4 summarizes FDA-approved monoclonal antibodies targeting EGFR and PD-1 receptors, including cetuximab, pembrolizumab, and nivolumab, along with their mechanisms of action, indications, trial phases, and common adverse effects. Table 5 presents EGFR-targeting TKIs, such as erlotinib, gefitinib, lapatinib, afatinib, and dacomitinib, which have been evaluated across multiple clinical trial phases [85,91]. For instance, in a multi-center Phase II trial (NCT00387127), lapatinib was compared to placebo in combination with cisplatin and radiotherapy in patients with unresected HNSCC [92]. The study evaluated complete response rates at 6 months post-chemoradiation and found that lapatinib was generally well tolerated, with numerical improvements in progression-free survival (PFS), particularly in p16-negative patients.

Similarly, a Phase II trial demonstrated that afatinib monotherapy was effective in recurrent or metastatic HNSCC resistant to platinum-based treatments, suggesting that sequential ErbB inhibition may benefit certain patient subgroups [85,92]. Despite afatinib’s clinical activity in HNSCC, its approval remains limited to EGFR mutation-positive NSCLC due to the lack of activating mutations in HNSCC [90,93]. Nevertheless, afatinib has shown promise in R/M HNSCC following platinum failure [85,90,94]. However, adjuvant afatinib after chemoradiotherapy (CRT) did not improve disease-free survival (DFS) and was associated with increased adverse events, making it unsuitable for routine use in primary unresected HNSCC [82,85,95,96].

The suboptimal response to EGFR inhibitors remains a major challenge in HNSCC [97]. To address this, emerging strategies are exploring combinations of TKIs with immunotherapy and radiotherapy. For example, the NIVOPOSTOP trial (NCT03576417) is a pivotal Phase III randomized clinical trial that evaluated the addition of nivolumab, an anti-PD-1 immune checkpoint inhibitor, to standard postoperative cisplatin-based chemoradiotherapy in patients with locally advanced HNSCC showing high-risk pathological features after surgery. The trial recently demonstrated that the addition of adjuvant nivolumab significantly improved the 3-year disease-free survival in high-risk, resected locally advanced HNSCC (63.1% vs. 52.5%; HR 0.76; *p* = 0.034), providing important clinical evidence supporting the integration of immunotherapy in the treatment of HNSCC [97].

Globally, 1266 clinical studies related to HNSCC were registered on ClinicalTrials.gov with start dates on or before 31 January 2022. Of these, 393 have been completed, while 590 are in various stages of recruitment. Despite the volume of research, a significant number of completed trials remain unpublished [98].

**Table 4:** Monoclonal antibodies target EGFR and PD-1 receptors, approved for treating HNSCC.

Medicine	Mechanism of Action	Year	Indication	Clinical Trial	Side Effect	Ref.
Cetuximab	Monoclonal antibody targeting EGFR	2006	HNSCC after platinum-based therapy (in combination with radiotherapy)	NCT00004227	Acneiform skin rash Fatigue Diarrhea Hypomagnesemia	[67]
		2009	R/M HNSCC (in combination with platinum–fluorouracil chemotherapy)	NCT00122460	Acneiform skin rash Fatigue Diarrhea Hypomagnesemia	[68]
Pembrolizumab	Monoclonal antibody targeting PD-1 receptor	2016	HNSCC after platinum-based chemotherapy	NCT01848834	Autoimmune toxicities Colitis Pneumonitis Thyroiditis	[99]
		2019	Metastatic or unresectable recurrent HNSCC (in combination with platinum and fluorouracil (FU) for all patients and as a single agent for patients whose tumors express PD-L1).	NCT02358031	Hepatitis Dermatitis Hypophysitis	[100]
		2025	Resectable locally advanced HNSCC (CPS ≥ 1); neoadjuvant + adjuvant ± cisplatin + RT	KEYNOTE-689	Pneumonitis, colitis, hepatitis, endocrinopathies	[55]
Nivolumab	Monoclonal antibody targeting PD-1 receptor	2014	R/M HNSCC with disease progression on or after a platinum-based therapy	NCT02105636	Autoimmune toxicities Colitis Pneumonitis Thyroiditis Hepatitis Dermatitis Hypophysitis	[101]

**Table 5:** EGFR-TKIs are approved for the treatment of HNSCC in clinical trials.

Medicine	MOA	Phase	Indication	Stage of Disease	Result	Ref.
Erlotinib	EGFR-TKI	I	Cissplatin+radiotherapy	Locally advanced	Safe combination	[102]
		II	Drug alone	Recurrent or metastatic	Stabilized disease	[87]
		I/II	Cissplatin + radiotherapy	Locally advanced	Feasible and well tolerated	[103]
Geftinib	EGFR-TKI	I	Cisplatin+radiotherapy	Locally advanced	Well tolerated with concomitant radiotherapy/chemoradiotherapy	[104]
		II	Drug alone	Recurrent/metastatic	Feasible and active	[105]
		III	Cisplatin + radiotherapy	Untreated/unresected/stage III/IV/nonmetastatic	Well tolerated	[106]
		II	Carbo/paclitaxel+RT	Locally advanced	Overall survival and complete response improvement	[107]
Lapatinib	EGFR-TKI	II	Cisplatin + radiotherapy	Locally advanced	Positive clinical activity was well tolerated	[92]
		II	Druga alone	Before chemoradiotherapy	Positive clinical activity, reduction, and cell proliferation index	[108]
Afatinib	EGFR-TKI	II	Compared with cetuximab	Metastatic and recurrent HNSCC	Sequential EGFR/ErbB treatment with afatinib and cetuximab provided sustained clinical benefits in patients after crossover, suggesting a lack of cross-resistance	[109]
Dacomitinib	EGFR-TKI	II	Drug alone	Metastatic and recurrent HNSCC	Well tolerated	[110]

MOA means mechanism of action; RT refers to radiotherapy.

## 6. Cannabidiol (CBD)

**Figure 3:**
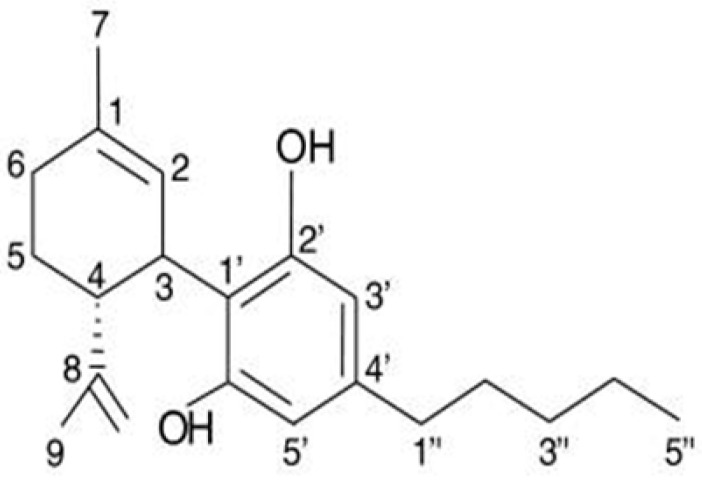
The chemical structure of cannabidiol (CBD) [118].

CBD (Figure 3) is a constituent of the *Cannabis sativa* L. (*marijuana*) plant family, and research on the chemical structure of CBD compounds has been ongoing since the 1960s. Cannabis plants contain two primary components, CBD and tetrahydrocannabinol (THC), each exerting distinct bioactive and medicinal effects on the human body [111].

In addition to its well-known antioxidant and immunomodulatory effects, CBD interacts with pathways in the body that are also involved in cancer progression. Accumulating evidence suggests that CBD demonstrates anti-tumor efficacy across various malignancies such as lung, breast, prostate, and colorectal cancer [112]. Studies using xenograft mouse models have shown that CBD can significantly reduce the migration, invasion, and viability of HNSCC cells in a dose- and time-dependent manner [113,114]. Additionally, CBD has demonstrated anti-tumor effects in HNSCC human cell lines and can enhance the efficacy of chemotherapy drugs [5]. CBD’s anticancer effects have been attributed to its interaction with the endocannabinoid system (ECS), apoptosis induction, and the suppression of oncogenic signaling [115].

The specific mechanism through which CBD reduces EGFR-mediated intracellular signaling is still not fully understood. The molecular structure of CBD is rich with aromatic rings and hydroxyl groups, which play crucial roles in establishing hydrophobic interactions and hydrogen bonds with numerous amino acids within the EGFR active site. Notably, CBD contains two hydroxyl groups. The significance of the phenolic hydroxyl groups in cannabinoids for both binding to and inhibiting EGFR-TK has been confirmed. CBD can be securely docked into the ATP-binding site of EGFR-TK through various weak interactions, as shown in Figure 3. These interactions include hydrophobic interactions among several amino acid residues and strong hydrogen bonds formed between a hydroxyl group of cannabinoids and Thr766, Gln767, and Met769 of EGFR-TK [116,117].

The combination of CBD with chemotherapeutic agents vinorelbine and 7-ethyl-10-hydroxycamptothecin has enhanced their efficacy in MCF7 breast cancer cells. A key molecular mechanism contributing to these synergistic effects is the enhancement of the pro-apoptotic activity of chemotherapeutic agents by CBD [119]. CBD’s induction of programmed cell death (apoptosis) was linked to the reduction in the levels of mTOR, AKT, 4EBP1, and cyclin D and the increased expression of PPAR-γ [9]. Moreover, CBD impeded the invasive and metastatic characteristics of aggressive triple-negative breast cancer (TNBC) by inhibiting the activation of the EGF/EGFR pathway and its downstream components (AKT and NF-κB). This was coupled with the downregulation of Id-1 protein by ERK and reactive oxygen species (ROS) [11]. Recent studies have attributed the anticancer activities of CBD to its binding to endocannabinoid receptors CB1 and CB2 [112], which subsequently render anti-inflammatory, pro-apoptotic, and antiproliferative effects [120].

### 6.1. Endocannabinoid Receptors (CB1 and CB2)

It is important to note that the effects of Δ9-THC (Delta-9-tetrahydrocannabinol) and cannabidiol depend on their ability to target specific endocannabinoid receptors, which belong to the G protein-coupled receptor family. CB1 receptors are primarily located in the central nervous system, particularly in the brain, while CB2 receptors are predominantly expressed in the immune system [118,121]. CBD has low affinity for CB1 and CB2 receptors compared to Δ9-THC, which is consistent with its lack of noticeable psychoactivity. Despite its low affinity towards CB1 and CB2 receptors, CBD can behave as a CB2 receptor inverse agonist [122], which binds as an agonist but inhibits CB2 receptors. Recent studies have linked cannabinoid receptors to the onset and advancement of tumors. The upregulation of CB1 or CB2 expression has been observed in hepatocellular carcinoma, renal cancer, and breast cancer. Moreover, this upregulation is correlated with the severity of the disease and a less favorable prognosis [123,124].

Nevertheless, conflicting functions have been documented for cannabinoid receptor pathways. While some studies suggest that cannabinoid receptors exhibit anti-tumoral effects, others demonstrate a tumor-promoting role for cannabinoids [8,125]. Changes in the endocannabinoid system (ECS) have been observed in breast cancer, notably with an increase in the expression of CB2 receptors. Specifically, research has found that over 90% of HER-2-positive tumors exhibit elevated expression of the cannabinoid receptor [126,127]. This overexpression of CB2 receptors is associated with a negative prognosis, likely due to the activation of HER2 pro-oncogenic pathways [126,128].

### 6.2. CBD as a Promising Anticancer Agent

It has been demonstrated that CBD has a major impact on cancer hallmarks through multiple pathways. The first pathway is the modulation of endoplasmic reticulum (ER) stress. ER homeostasis refers to the balance between the protein load within the ER and its capacity to properly fold proteins. This balance can be disrupted by various physiological conditions, leading to protein folding demand and folding capacity. As a result, unfolded proteins accumulate, inducing ER stress [129]. Subsequently, unfolded protein response (UPR) is activated in response to ER stress, leading to the activation of three parallel pathways, which are protein kinase RNA-like ER kinases (PERK), activating transcription factor 6 (ATF6), and inositol-requiring enzyme 1 (IRE-1) [130]. In a cancer environment, ER stress and UPR have a distinguishable function in malignancy development. This results in malignant survival, oncogenic transformation, and cancer progression. CBD is a potent ER stress inducer, which causes prolonged toxic signals to induce apoptosis in cancer cells [129,131]. For example, the controlled exacerbation of pre-existing ER stress in the cancer cell can overload the ER stress scheme, which leads to the stimulation of the pro-apoptotic component of the UPR [132].

Another possible mechanism of CBD in malignancy is the modulation of major cell cycle pathways. In a notable study on gastric cancer cells, CBD was reported to induce cell cycle arrest at the G0–G1 phase, which was associated with a reduction in CDK2/cyclin E protein levels [133]. Addressing local recurrence and the metastasis of cancer remains a contemporary challenge in the treatment of HNSCC. To combat this issue, the application of combination therapy containing drugs with multiple anticancer mechanisms has been proposed as a potential therapeutic strategy to overcome multidrug resistance and to improve therapeutic efficacy [134].

In addition, the role of classical cannabinoid receptors in the progression of HPV-positive HNSCC has been investigated. The study revealed that the knockdown of CB1 and CB2 receptors suppressed the growth of HPV-positive HNSCC cells, whereas activation with CB1/CB2 agonists increased proliferation and migration and reduced apoptosis. These effects occurred via the specific activation of the p38 MAPK pathway. Moreover, increased p38 MAPK activation has been observed in HPV-positive HNSCC patients exposed to cannabinoids [135]. However, the role of cannabinoids in the HPV-positive HNSCC microenvironment remains largely unexplored. A recent preclinical study provided direct evidence that CBD suppresses HPV-positive HNSCC by promoting apoptosis, activating MAPK signaling (ERK1/2, JNK/SAPK, and MK2), and enhancing anti-tumor immunity through CD4^+^/CD8^+^ T-cell infiltration and activation, particularly in immune-competent models [136].

Hijiya et al. (2017) have demonstrated an independent association between CB1 overexpression and an unfavorable prognosis in esophageal SCC [137]. Additionally, in another study, the high expression of CB2 in HNSCC has been correlated with reduced disease-specific survival [138]. On the contrary, some researchers found elevated levels of CB1 and CB2 in mobile tongue SCC, and this heightened expression was associated with a more favorable prognosis [139]. Further mechanistic studies are warranted to elucidate the mechanisms underlying such discrepancy between clinical association studies.

Research has explored the anti-angiogenic properties of CBD using human umbilical vein endothelial cells. The findings indicate that CBD causes endothelial cells to cease proliferation without triggering cell death and effectively reduces their ability to migrate, invade, and form new blood vessel structures both in vitro and in vivo. These actions are associated with reduced levels of several key angiogenesis-related molecules, such as matrix metalloproteinases MMP2 and MMP9, urokinase-type plasminogen activator (uPA), endothelin-1 (ET-1), platelet-derived growth factor-AA (PDGF-AA), and the chemokine CXCL16 [15], as seen in Table 6, which shows the mechanism of action of CBD, molecular targets, and biological effects.

CBD has been shown to enhance chemosensitivity and reverse drug resistance in HNSCC models. Go et al. (2020) reported that CBD significantly reduced cell viability, migration, and invasion in various HNSCC cell lines and xenograft models. When combined with chemotherapeutics, like fluoropyrimidines, platinum analogs, or taxanes, CBD exhibited synergistic cytotoxic effects. Mechanistically, CBD induced apoptosis and autophagy while downregulating key genes involved in DNA repair, cell proliferation, and cell cycle progression, which are related to therapeutic resistance in HNSCC. Furthermore, CBD modulates signaling pathways, such as PI3K/AKT/mTOR and MAPK/ERK, which are often dysregulated in HNSCC, thereby overcoming survival mechanisms and enhancing drug efficacy [5].

### 6.3. Synergism Between CBD and TKIs on EGFR

As mentioned previously, EGFR is overexpressed in many types of cancer, including HNSCC. Experimental data have shown that CBD can inhibit EGF-induced EGFR-TK activity, leading to the suppression of tumor cell growth and the induction of apoptosis in vitro and in vivo [11]. CBD has demonstrated a high binding affinity for purified EGFR-TK and a potent inhibitory effect against EGFR-TK, with IC50 values of 32 nM (compared with an IC50 value of 3 nM for afatinib), which is consistent with the in silico characterization of molecular interactions between CBD and EGFR-TK [14]. Gefitinib exhibited enhanced therapeutic efficacy and reduced cytotoxicity when combined with CBD [14]. These results provide strong support for the development of CBD as a potential adjunct to EGFR TKI-based treatment options in patients with EGFR-positive cancers.

Importantly, EGFR activation can happen via transactivation by other receptors and mediators. For example, human corneal epithelial cell (HCEC) proliferation and migration are enhanced in response to CB1 and transient receptor potential cation channel vanilloid member 1 (TRPV1) activation—targets of CBD—which transactivate EGFR and subsequently trigger the MAPK and Akt/PI3K pathways [12,13]. CBD has been shown to directly stimulate the TRPV1 receptor, which triggers intracellular signaling through the influx of calcium. Capsaicin, a model TRPV1 agonist, leads to apoptosis induction through mitochondrial membrane depolarization, the generation of ROS, and the activation of caspases 9 and 3 [140]. Similarly, CBD increased ROS production, mediated by elevated intracellular Ca^2+^ [141], which results in altering the mitochondrial membrane potential and causes the production of ROS in MDA-MB-231 cells [141]. TRPV1-mediated release of cytokine mediators, such as interleukin (IL)-6 and the chemoattractant (IL-8), is achieved via both the EGFR-dependent and EGFR-independent signaling pathways [142]. These examples highlight the complexity of CBD-EGFR interactions.

## 7. Conclusions

This review explores the molecular rationale for combining CBD with EGFR TKIs in the treatment of HNSCC. Despite promising preclinical evidence demonstrating CBD’s anticancer and immunomodulatory effects, no clinical data currently support its use as an adjunct to EGFR-TKIs in HNSCC; thus, this remains a hypothesis requiring further investigation. Significant knowledge gaps exist regarding how CBD interacts with dysregulated signaling pathways in HNSCC in the presence and absence of an EGFR-TKI. Future research should focus on elucidating these mechanisms through rigorous in vitro and in vivo studies. Testing this hypothesis is critical, as combining CBD with EGFR-TKIs could lay a transformative foundation for significantly enhancing treatment efficacy and patient outcomes in HNSCC, potentially converting a suboptimal targeted therapy into a highly effective therapeutic strategy. Further research is warranted to establish greater confidence in supporting experimental and clinical correlative data and address key gaps in current knowledge.

## Figures and Tables

**Table 1:** Histopathological and molecular progression of OSCC [23,46,47].

Histopathological Stage	Key Molecular Events
Hyperplasia	p16 deletion, EGFR overexpression
Mild to moderate dysplasia	*TP53* mutation, p21 deletion
Carcinoma in situ	Cyclin D1 amplification, chromosomal deletions at 11q24 and 11q31
Invasive SCC	*PTEN* inactivation, deletions at 18q and 4q26–28, loss of E-cadherin

**Table 2:** A summary of target therapies for EGFR in the treatment of HNSCCs [53].

Mechanism of Action	Molecularly Targeted Therapy Drug
EGFR monoclonal antibodies	Cetuximab, panitumumab, zalutumumab, and nimotuzumab
EGFR–tyrosine kinase inhibitors	Gefitinib, erlotinib, lapatinib, afatinib, and dacomitinib Sorafenib, sunitinib, and vandetanib

**Table 3:** A summary of EGFR mutations across NSCLC vs. HNSCC, with prevalence and TKI sensitivity.

EGFR Exon	Common Mutation(s)	Prevalence in HNSCC	Prevalence in NSCLC	TKI Sensitivity	References
Exon 18	G719S/C/A	Rare	~3–5%	Sensitizing to 1st- and 2nd-gen TKIs	[75]
Exon 19	Del E746-A750	Rare	~45%	High sensitivity to erlotinib and gefitinib	[75,76,78]
Exon 20	Insertions	Rare	~4–10%	Generally resistant to 1st/2nd-gen TKIs	[75]
Exon 21	L858R	Rare	~40%	High sensitivity to erlotinib and gefitinib	[75,76]

**Table 6:** CBD’s mechanism of action, molecular targets, and biological effects.

Mechanism of Action	CBD Target	Biological Effect	Reference(s)
**Endoplasmic** **reticulum (ER) stress**	Induces ER stress; activates UPR (PERK, IRE1, ATF6)	Triggers apoptosis via prolonged ER stress	[129,130,131,132]
**Cell cycle regulators**	Downregulates CDK2 and cyclin E	Causes G0/G1 cell cycle arrest; inhibits proliferation	[133]
**Apoptosis and autophagy**	Induces caspase activation; promotes autophagy	Initiates programmed cell death and autophagy	[5]
**PI3K/AKT/mTOR pathway and EGFR signaling**	Downregulates mTOR, AKT, 4EBP1, cyclin D; inhibits EGF/EGFR and NF-κB pathways	Promotes apoptosis; inhibits invasion and metastasis	[9,11]
**MAPK/ERK pathway**	Modulates ERK1/2, JNK/SAPK, MK2	Promotes apoptosis; enhances anti-tumor immunity	[135,136]
**Cannabinoid receptors (CB1/CB2)**	Binds with low affinity; acts as a CB2 inverse agonist	Modulates proliferation, apoptosis, and immune responses	[8,112,120,122,125]
**Angiogenesis-related molecules**	Reduces MMP2, MMP9, uPA, ET-1, PDGF-AA, CXCL16	Inhibits angiogenesis, migration, and invasion	[15]
**DNA repair and cell proliferation genes**	Downregulates genes involved in DNA repair and cell growth	Sensitizes cancer cells to therapy; overcomes resistance	[5]
